# Transcriptomic Analysis of Purified Embryonic Neural Stem Cells from Zebrafish Embryos Reveals Signaling Pathways Involved in Glycine-Dependent Neurogenesis

**DOI:** 10.3389/fnmol.2016.00022

**Published:** 2016-03-31

**Authors:** Eric Samarut, Abdelhamid Bekri, Pierre Drapeau

**Affiliations:** ^1^Department of Neurosciences, Research Center of the University of Montreal Hospital CenterMontréal, QC, Canada; ^2^Department of Biochemistry and Molecular Medicine, University of MontréalMontréal, QC, Canada

**Keywords:** neurogenesis, stem cell, interneuron, glycine, zebrafish

## Abstract

How is the initial set of neurons correctly established during the development of the vertebrate central nervous system? In the embryo, glycine and GABA are depolarizing due the immature chloride gradient, which is only reversed to become hyperpolarizing later in post-natal development. We previously showed that glycine regulates neurogenesis *via* paracrine signaling that promotes calcium transients in neural stem cells (NSCs) and their differentiation into interneurons within the spinal cord of the zebrafish embryo. However, the subjacent molecular mechanisms are not yet understood. Our previous work suggests that early neuronal progenitors were not differentiating correctly in the developing spinal cord. As a result, we aimed at identifying the downstream molecular mechanisms involved specifically in NSCs during glycine-dependent embryonic neurogenesis. Using a *gfap:GFP* transgenic line, we successfully purified NSCs by fluorescence-activated cell sorting from whole zebrafish embryos and in embryos in which the glycine receptor was knocked down. The strength of this approach is that it focused on the NSC population while tackling the biological issue in an *in vivo* context in whole zebrafish embryos. After sequencing the transcriptome by RNA-sequencing, we analyzed the genes whose expression was changed upon disruption of glycine signaling and we confirmed the differential expression by independent RTqPCR assay. While over a thousand genes showed altered expression levels, through pathway analysis we identified 14 top candidate genes belonging to five different canonical signaling pathways (signaling by calcium, TGF-beta, sonic hedgehog, Wnt, and p53-related apoptosis) that are likely to mediate the promotion of neurogenesis by glycine.

## Introduction

Neurogenesis is a crucial step in vertebrate development and it requires multiple and integrated signals throughout development to be achieved correctly (Urban and Guillemot, 2014; Mitrousis et al., 2015). Many factors have already been identified as regulators of neurogenesis in the spinal cord (Jessell, 2000) and glycine signaling has been recently shown to be one of those (McDearmid et al., 2006). In addition to being the smallest and the simplest of the essential amino acids, glycine is also a neurotransmitter involved in inhibitory circuits. With gamma-aminobutyric acid or GABA, they are the two major inhibitory neurotransmitters in the central nervous system, with glycine acting mainly in the brainstem and in the spinal cord.

During development, prior to glutamatergic innervation, neural activity mainly relies on glycinergic and GABAergic neurotransmission (Ben-Ari, 2002). Interestingly, although these neurotransmitters are mostly inhibitory in the adult neural system, they elicit an excitatory activity in the early embryo. Indeed, contrary to their hyperpolarizing role in adult neurons, GABA and glycine result in a chloride-induced membrane depolarization in the embryo, which generates the earliest forms of electrical signaling in the immature nervous system. This switch in the electrical activity of these neurotransmitters has been correlated with the late expression of the neuron-specific and extrusive chloride/potassium co-transporter2 (KCC2), which decreases chloride load in mature neurons and therefore underlies the hyperpolarisation of the cell when the glycine receptors (GlyRs) open. In contrast, in embryonic neurons the chloride load is higher and glycine binding to GlyRs provokes a release of chloride ions and therefore induces a depolarization of the cell. This excitatory effect of glycine during embryonic development is necessary for a broad range of neurogenic processes from formation to maturation of neuronal circuits (Ben-Ari, 2002). Of particular interest, mutations of glycine signaling, such as mutations in GlyRs (Pilorge et al., 2015) and KCC2 (Merner et al., 2015), have recently been implicated in autism, arguably impacting on neurogenesis.

Previously, our group discovered an early role of glycine during neurogenesis in the spinal cord, which starts during the first day post-fertilization (dpf), prior to hatching after 2 dpf. Glycine acts to promote the differentiation of neural progenitors specifically into interneurons (McDearmid et al., 2006) in a process that is impeded upon KCC2 expression (Reynolds et al., 2008). More recently, we showed that paracrine release of glycine is necessary and sufficient to drive correct interneuron differentiation (Cote and Drapeau, 2012) and that this involves calcium transients in neural progenitors (Brustein et al., 2013). Finally, our previous work showed that unlike in wild type embryos, proliferating neural progenitors in the spinal cord of embryos in which glycine signaling is impaired are still very numerous at later stages of development (i.e., 3 dpf) as they fail to differentiate (McDearmid et al., 2006; Cote and Drapeau, 2012). As a result, we concluded that glycine signaling is most likely to play a role in regulating the differentiation of early neural progenitors or neural stem cells (NSCs) into interneurons during spinal cord development.

Although the requirement for glycine signaling for interneuron neurogenesis is now well established, the subjacent molecular mechanisms are not yet understood. In this work, we aimed at shedding light on the molecular action of glycine signaling during neural development and to identify the downstream molecular pathways involved. We took advantage of a transgenic line in which NSCs are fluorescently labeled in order to develop a protocol to specifically purify the NSC population from whole embryos by fluorescence-activated cell sorting (FACS). After mRNA purification, we performed next generation sequencing to access the transcriptome of NSCs purified from embryos in which glycine signaling had been disrupted by morpholino knockdown. This allowed us to compare the set of genes differentially expressed in NSCs purified from *glra4a* morpholino-injected versus control morpholino-injected and non-injected zebrafish embryos and we confirmed their differential expression by independent RTqPCR assay. Using pathway analysis and clustering tools, we identified several canonical signaling pathways involved in glycine-dependent neurogenesis.

## Materials and Methods

### Fish Husbandry

Wild-type zebrafish (*Danio rerio*) were reared at 28.5°C, kept under a 12-h dark, 12-h light cycle, and staged as described previously (Kimmel et al., 1995). They were bred according to standard procedures (Westerfield, 1995). All experiments were performed in compliance with the guidelines of the Canadian Council for Animal Care and conducted at the Research Center of the University of Montreal Hospital Center (CRCHUM).

### Microinjection

A morpholino oligonucleotide targeting the *glra4a* GlyRs (McDearmid et al., 2006) (using new GlyR nomenclature) was purchased from Gene-Tools and resuspended in ultrapure water at 1 mM stock solution as recommended. A total of 0.6 ng of morpholino was injected at the one-cell stage (5′-TGATAATGAGAGAGAAATGCGTCA-3′; ENSDART00000026194). For control knockdown, the same morpholino but harboring five base mismatches was ordered and injected accordingly (5′-TcATAATGAcAcAGAAATcCGTgA-3′; referred as control morpholino).

### Cloning of Reporter Constructs

Total RNA was isolated from 24 hpf zebrafish embryos using TRIZOL^®^ reagent. 1 μg of total RNA was reverse transcribed into cDNA using VILO reverse transcription mix (Invitrogen). Primers were designed to flank the part of the 5′UTR/5′CDS of zebrafish *glra4a* gene containing the morpholino binding site targeted in this study. The resulting 0.1 kb segment was sub-cloned in-frame into pEGFP-N1 to generate a fusion protein (primer sequences are available upon request). Thirty nanogram of *glra4a*-EGFP plasmid was microinjected with 0.6 ng of *glra4a* or control morpholino, respectively. The fluorescence was analyzed by confocal microscopy (Olympus BX61W1 equipped with a Quorum Technology spinning-disk confocal head) at 18 hpf.

### Single-Cell Dissociation and FACS

Embryos at 20 hpf were briefly washed in calcium-free Ringer’s solution and deyolked by up and down pipetting. Deyolked embryos were pelleted by 500 ×*g* centrifugation for 5 min. They were briefly washed with FACSmax cell dissociation solution (Genlantis) and transferred in a 60 mm petri dish with fresh FACSmax solution, then incubated at 28.5°C. Single-cell dissociation was carefully monitored every 5 min and was generally achieved within 30 min of incubation. Efficient dissociation was helped by firmly tapping the petri dish and by gentle pipetting. Single cells were exhaustively washed twice in cold PBS, pelleted and resuspended in 500 μL of cold PBS. Single cells were filtered in a Falcon tube with a cell strainer cap (Fisher Scientific) and placed on ice until cell sorting. Sorting was performed using a BDARIA IIIu FACS with DIVA 8 sofware (BD Biosciences San Jose, CA, USA). GFP expressing cells were identified using a 488 nm laser and a 530/30 BP filter. Cells were collected in cold PBS on ice.

### Transcriptomic Assay, Differential Expression Assay, and Pathway Analysis

Two independent clutches were injected, by each of two experimenters, corresponding to experimental duplicates. Total RNA from RNAlater-fixed embryos (Ambion) was extracted using RNAsolv reagent (Omega Biotek) following the manufacturer’s standard protocol. RNA extraction was made using between 5.19–9.75 × 10^5^ cells by RNAsolv reagent manufacturer’s protocol (Omega Biotek). Absence of contamination with chemicals was assessed by nanodrop using 260/280 and 260/230 ratios. Quantification of total RNA was made by nanodrop and 0.1 to 1.44 g of total RNA was used for sequencing. Quality of total RNA was assessed with the BioAnalyzer Nano (Agilent) and all samples had a RNA Integrity Number (RIN) above 8.

Library preparation was done with the Truseq RNA (Illumina). 18 PCR cycles was required to amplify cDNA libraries. Libraries were quantified by nanodrop and BioAnalyzer. All librairies were diluted to 10 nM and normalized with the Miseq SR50 v2. Libraries were pooled to equimolar concentration and multiplexed by six samples per lane. Sequencing was performed with the Illumina Hiseq2000 using the SBS Reagent Kit v3 (100 cycles, paired-end) with 1.6 nM of the pooled library. Cluster density was targeted at around 800 k clusters/mm^2^. Around 65 to 89 million reads were generated for each sample. Library preparation and sequencing was done at the Institute for Research in Immunology and Cancer’s Platform (University of Montreal). About 86% of high quality reads were mapped onto the zv9 version of the zebrafish genome (ensemble release 77) using TopHat version 2.0.10.

Differential gene expression analysis was assessed by DeSeq2 package using R software. Differential gene expression was filtered on an absolute value of LogFC > 1 and a False Discovery Rate (or adjusted *p*-value) < 0.05. Pathway analysis was performed using DAVID bioinformatics resources (Huang da et al., 2009). The list of differentially expressed genes was uploaded onto DAVID analysis wizard and a list of all expressed genes found in our dataset was used as a background for gene enrichment analysis. We also benefitted form a free trial of the Ingenuity Pathway Analysis software from QIAGEN. Pathway information was generated using the Kyoto Encyclopedia of Genes and Genomes (KEGG: www.genome.jp/kegg/pathway).

### RT-Quantitative PCR (RTqPCR)

Quantitative PCR (qPCR) primers for each tested gene were designed using the Universal Probe Library tool from Roche. Total RNA was extracted from FACS-purified NSCs using RNAsolv reagent (Omega Biotek) and chloroform followed by isopropanol precipitation. Reverse transcription was performed from 1 μg of total RNA using the superscript VILO reverse transcription mix (Invitrogen). Quantitative PCR was performed on 2 μL of 1:10-diluted cDNA using SYBR Green I master (Roche) on a LightCycler 80 thermocycler. *polr2d* gene (ENSDART00000108718) was used as a reference gene for ddCt quantification

## Results

### FACS-Purification of NSCs from Whole Zebrafish Embryos

**FIGURE 1:**
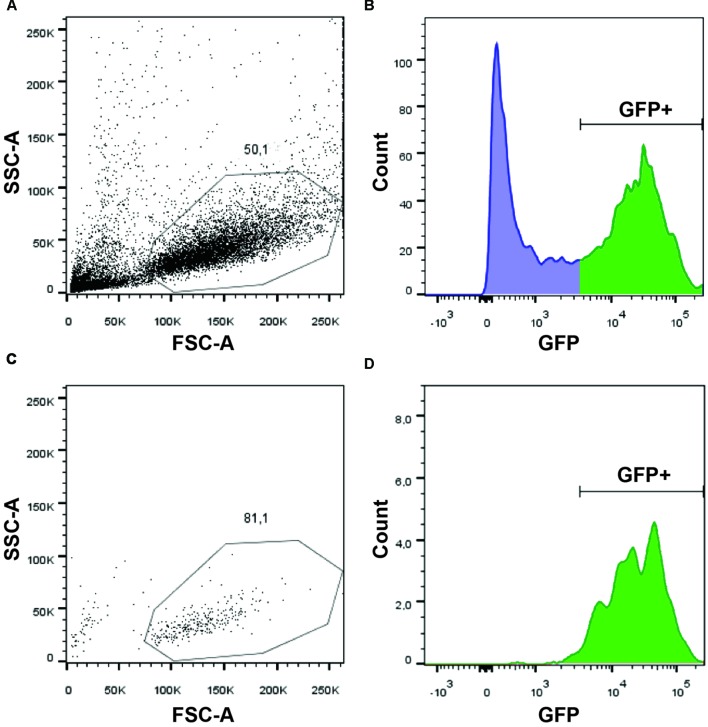
**Purification of GFP+ NSCs by FACS.** Dissociated cells were analyzed on a BDARIA IIIu FACS and visualized according to their relative size (FCS, Forward Scatter) and granularity (SSC, Side Scatter) before **(A)** and after **(C)** cell sorting. About 50% of the events were dissociated cells suitable for sorting. GFP expressing cells were easily identifiable using a 530/30 filter **(B)**. Sorted cells were analyzed post-sorting to confirm the selective and specific sorting of GFP+ cells **(D)**.

In order to identify the signaling pathways involved in glycine-mediated NSC differentiation, we took advantage of the *gfap:GFP* transgenic line that has been reported to express GFP in NSCs (Lam et al., 2009). Using the *mi2001* allele (Bernardos and Raymond, 2006), we were able to detect, as reported, fluorescence in the spinal cord from 20 hpf, near the beginning of neurogenesis and onward. Since we aimed at studying the transcriptomic changes induced by GlyR knockdown specifically in NSCs, we set up a protocol to purify this population of cells from whole zebrafish embryos. As a result, we conserved the integrity of the embryonic *in vivo* context but *in fine* we only focused on our cells of interest. Using a protease cocktail (see Materials and Methods), we successfully dissociated 20 hpf embryos into single cells and we were able to perform a FACS purification of GFP+ cells (e.g., NSCs) (**Figure 1**). As shown in **Figure 1A**, the quality of the single-cell dissociation was suitable for subsequent FACS with more than 50% healthy single cells. After doublet elimination and using a 530/30 BP filter, the GFP+ population (i.e., NSCs) was easily distinguishable from the negative batch as separate peaks in the cell count (**Figure 1B**). In order to prevent any contamination with GFP- cell types, we increased the stringency of the sorting by only selecting cells with a high GFP expression (>3,000 fluorescence units). Finally, to ensure the specificity of the sorting and the integrity of the purified cells, we performed a quality control by further analyzing the GFP+ sorted cells (**Figures 1C,D**). We found that about 81% of cells survived and more than 97% of them were GFP+. Together these results indicate that live NSCs can indeed be purified by FACS and so used to explore the neurogenic role of glycine.

### Glycine Receptor Knockdown NSCs

**FIGURE 2:**
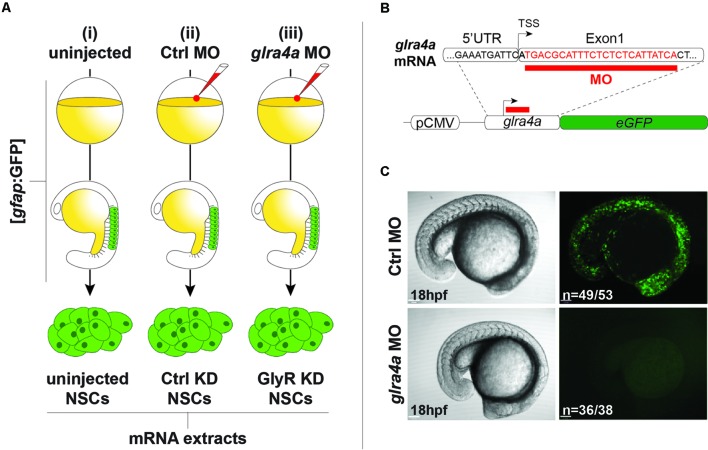
**Glycine receptor (GlyRs) knock-down NSCs. (A)** Experimental protocol to purify NSCs from (i) uninjected, (ii) Ctrl MO-injected, and (iii) glra4a MO-injected. *gfap*:GFP embryos were injected at the one-cell stage and let develop until 20hpf. NSCs were sorted by FACS and harvested for total RNA extraction. **(B)** *glra4a* morpholino targets 24 nucleotides (in red) downstream to the transcription starting site. A 100 bp fragment encompassing the end of the 5UTR and the 5 part of the first exon was cloned in frame with eGFP coding sequence downstream to the CMV promoter. **(C)** Embryos co-injected with the *glra4a-eGFP* construct and Ctrl morpholino depict broad GFP fluorescence at 20 hpf (upper right). However, co-injection with *glra4a* morpholino abolishes fluorescence (lower right) thus validating the use of both Ctrl and *glra4a* morpholinos.

We used the same procedures to purify NSCs from (i) uninjected 20 hpf embryos, (ii) embryos injected with a 5 base-mismatch control morpholino, and (iii) embryos knocked-down for GlyR using a specific *glra4a* morpholino (**Figure 2A**). To validate the *in vivo* efficacy and selectivity of our morpholino, we cloned a 0.1 kb fragment of the *glra4a* region containing the morpholino target site (in red in **Figure 2B**) in frame with the coding sequence of GFP. After co-injection of this construct and our control morpholino, 92% of the embryos (*n* = 49/53) depicted broad GFP fluorescence, thus validating the expression of the fusion protein (**Figure 2C**). However, when co-injected with *glra4a* morpholino, GFP was undetectable in 36 out of 38 of the injected embryos (95%). These results validate the selectivity of our *glra4a* morpholino compared to the 5 base-mismatch control morpholino. For each condition (uninjected, control, and GlyR knockdown), we dissociated between 60 and 80 *gfap:GFP*^+^ embryos from which we successfully FACS-purified between 5.19 and 9.75 × 10^5^ GFP+ cells (**Figure 2A**). Each treatment condition was processed in duplicate from independent batches of eggs and total RNA was extracted reaching a RIN higher than 8.50.

### Deep-Sequencing and Differential Expression Analysis of Purified NSCs

**FIGURE 3:**
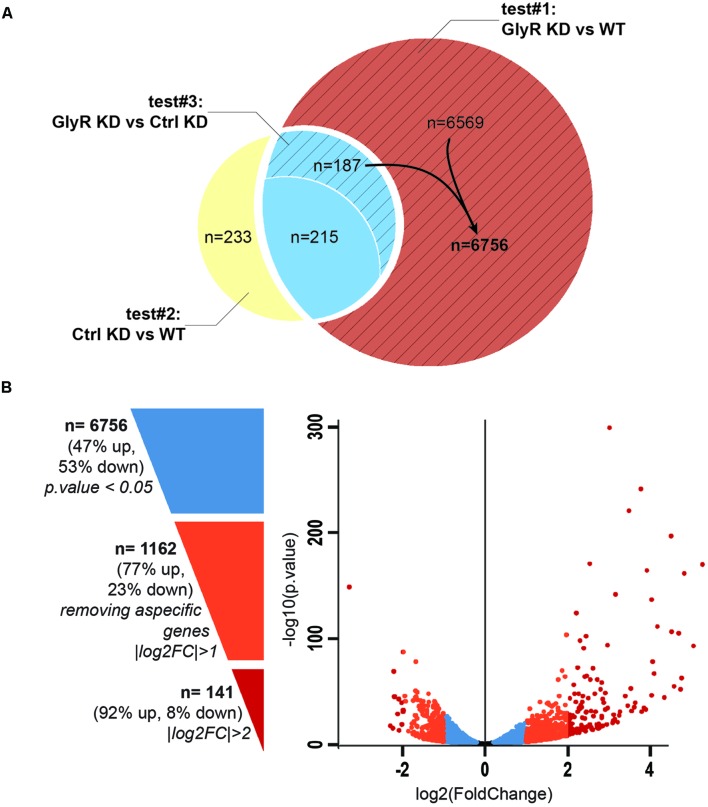
**Differential expression analysis. (A)** Venn diagram showing the total number of differentially expressed genes in each tests. Test#1 identified a total of 6971 differentially expressed genes (6569 + 187 + 215, *p*-value < 0.05) by comparing the transcriptome of NSCs from *glra4a* morpholino-injected (GlyR KD) versus uninjected embryos (WT). Test#2 identified 635 differentially expressed genes between the transcriptome of NSCs from control morpholino-injected (Ctrl KD) versus uninjected (WT) (233 + 215 + 187, *p*-value < 0.05). 402 genes (215 + 187) were overlapping between tests #1 and #2 (in blue). Test#3 compared the transcriptome of GlyR KD versus Ctrl KD for these 402 genes and identified 187 genes for which the differential expression was significant (*p*-value < 0.05). These genes were compiled with 6569 genes differentially expressed in GlyR KD (in red) and compose the final list of 6756 specific genes differentially expressed in NSCs upon GlyR KD (stripped red). **(B)** Volcano plot showing each of the 6756 genes from the final list established above. Blue dots represent the total 6756 specific genes filtered on a *p*-value < 0.05. Orange dots show the 1162 genes with a |log2FC| > 1 and red dots show the 141 genes with a |log2FC| > 2.

Between 65 and 89 million high quality reads per condition were sequenced and we were able to map about 86% of these reads onto the zv9 version of the zebrafish genome (ensemble release 77). Using DESeq2 algorithm, we assessed the differentially expressed genes comparing the dataset from GlyR knockdown NSCs with uninjected WT NSCs (test#1, **Figure 3A**) and found 6,971 differentially expressed genes (*p*-value < 0.05).

We assumed that some of these genes could be misregulated because of the injection itself rather than being specific to the GlyR knockdown. To circumscribe this issue, we therefore performed a differential gene expression analysis in NSCs from control-morpholino injected embryos compared to uninjected (test#2). We found that 635 genes were misregulated in NSCs upon injection of the control morpholino (the full list of these genes is available upon request). Moreover, 402 of them were overlapping with the list of genes found to be differentially expressed upon GlyR knockdown (in blue in **Figure 3A**). Most likely, we assume that these genes (6%) are morpholino off-targets and were removed for further analysis. To this aim, we also ran a third differential analysis test (#3) comparing the GlyR knockdown dataset with control-morpholino knockdown. This analysis revealed that the expression of 215 genes from this overlapping list were not statistically different between GlyR and control knockdown. Thus, we considered these genes as off-targets and we removed them as well from the final list. However, we kept those genes whose differential expression between GlyR and control knockdown was significant (*p*-value < 0.5) since although being found in the control condition, the difference of expression for these genes seemed to be specific to GlyR knockdown (stripped blue in **Figure 3A** and highlighted in blue in **Supplementary Table S1**).

We concluded that a total of 6,756 genes were specifically and differentially expressed in NSCs upon GlyR knockdown (*p*-value < 0.5) and this number decreases to 1,162 after filtering with a |logFC| greater than one (at least 2 fold up- or down-regulation; **Supplementary Table S1** and **Figure 3B**). As visualized in the volcano plot (**Figure 3B**), 77% of these genes were upregulated upon GlyR knockdown (895/1,162; green arrow up in **Supplementary Table S1**) and 23% were downregulated (267/1,162; red arrow down in **Supplementary Table S1**). By filtering more stringently with a |logFC| > 2 (at least 4 fold up- or down-regulation), we could narrow down this number to 141 genes (dark red in **Figure 3B**).

### Identification of Pathways Involved in Glycine-Dependent Neurogenesis

**FIGURE 4:**
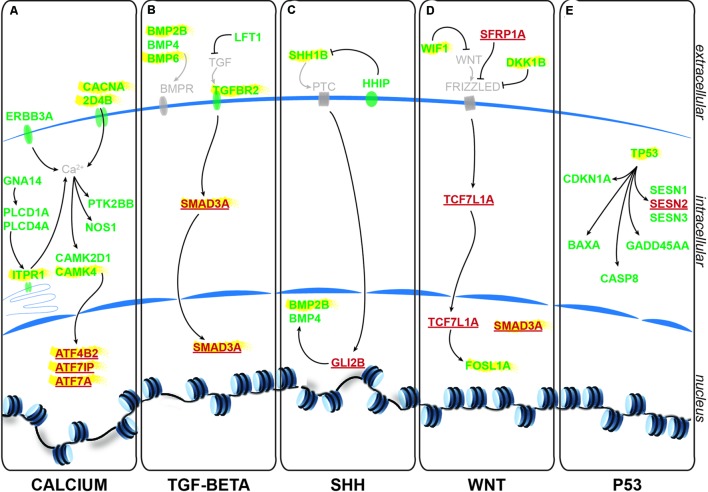
**Identified signaling pathways downstream to glycine in NSCs.** Representation of the pathways identified and of the key genes involved according to KEGG and IPA. For each signaling pathway **(A)** calcium, **(B)** TGF-beta, **(C)** SHH, **(D)** WNT, and **(E)** P53, upregulated genes upon GlyR KD are noted in green and downregulated genes are in red and underlined. Genes highlighted in yellow have been tested by RT-qPCR to confirm RNA-sequencing data (see **Figure 5**).

Using the freely available DAVID bioinformatics resources (Huang da et al., 2009) and through orthologous extrapolation from the IPA software (Ingenuity Pathway Analysis, QIAGEN) we performed pathway analysis using our list of 1,162 specific genes differentially expressed in NSCs upon GlyR knockdown. To this aim, we used the KEGG annotation of identified pathways involving genes of our list. Interestingly, five main molecular pathways came out from this analysis as being enriched in our dataset (**Figures 4A–E**): signaling by (i) calcium, (ii) TGF-beta, (iii) sonic hedgehog, (iv) Wnt, and (v) p53-related apoptosis. Regarding calcium signaling, we found CACNA2D4B and other calcium channels (CACNG1, CACNG8B, and CACNB2B) to be upregulated. Consistently, upon GlyR knockdown, the ITPR1 intracellular calcium channel expressed at the endoplasmic reticulum was also upregulated. Besides, the expression of genes encoding the Gαq GNA14 and the downstream phospholipases C (PLCD1A/4A), which activate ITRP1, were also upregulated in our dataset. Interestingly, we found that some calcium-dependent proteins such as calmodulin kinases CAMK2D1 and CAMK4, the proline-rich tyrosine kinase PTK2BB and the nitric oxide synthase 1 NOS1 were upregulated in NSCs upon GlyR knockdown. Finally, some transcription factors regulated by calmodulin kinases and belonging to the CREB complex were downregulated in our dataset (ATF4B2, ATF7IP, and ATF7A) (**Figure 4A**).

Furthermore, we found some genes involved in the Transcription Growth Factor TGF-beta signaling pathway such as Bone Morphogenetic Proteins (BMP) 2B, 4 and 6, TGF-beta receptor 2 (TGFBR2) and the TGF inhibitor Lefty1 (LFT1) as upregulated in our knockdown dataset (**Figure 4B**). In the same pathway, the transcription factor SMAD3A was found to be downregulated in response to GlyR knockdown in NSCs.

According to DAVID ressources, BMP2B and BMP4 are also involved in Sonic HedgeHog signaling (SHH) (**Figure 4C**) in which they are activated by GLI2B zinc-finger transcription factor (GLI-Kruppel family member 2B) and the expression of this gene was downregulated in our GlyR knockdown dataset. We also observed the upregulation of SHH1B gene itself and of the SHH interacting protein HHIP.

The fourth pathway identified is the WNT signaling pathway in which two inhibitor proteins WIF1 (Wnt inhibitory factor 1) and DKK1B (Dickkopf wnt inhibitor 1B) were upregulated upon GlyR knockdown (**Figure 4D**). Moreover, we found that the secreted frizzled-related protein 1a (SFRP1A) and the target transcription factor TCF7L1A were downregulated after GlyR knockdown. On the contrary, the expression of the leucine zipper FOS-like antigen 1A (FOSL1A) was upregulated in our dataset.

Lastly, we found p53 itself and numerous downstream targets of the p53-related apoptosis pathway (SESN1 and 3, CDKN1A, BAXA, GADD45AA, and CASP8) to be activated upon GlyR knockdown, while SESN2 was downregulated (**Figure 4E**).

### Validation of Transcriptomic Results by RTqPCR

**FIGURE 5:**
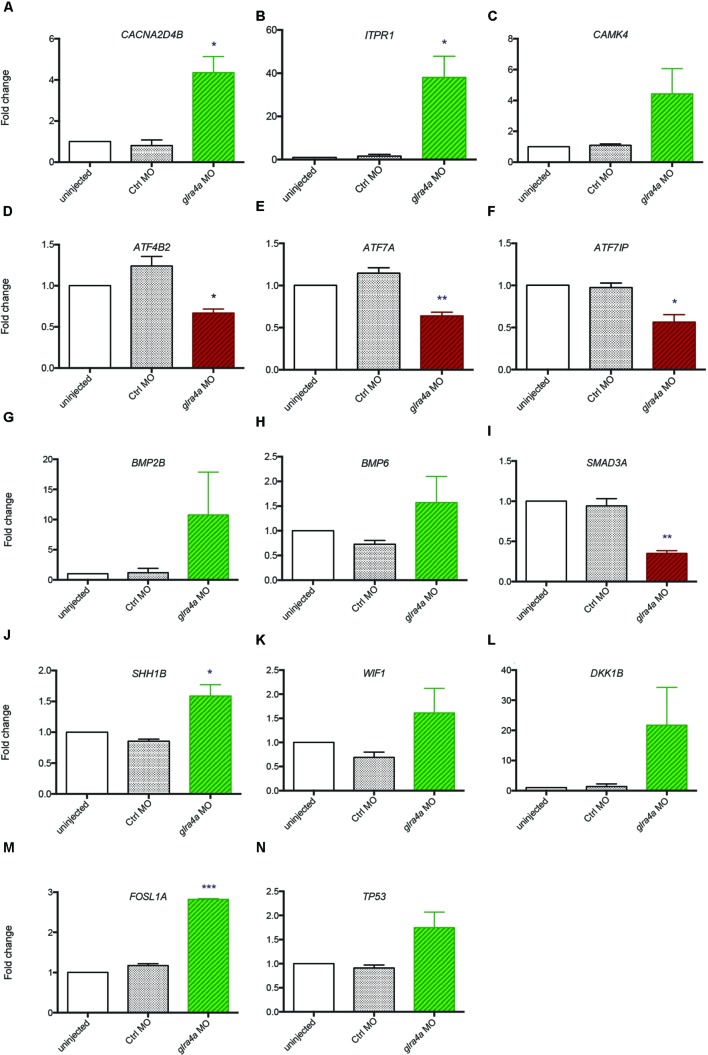
**Validation of transcriptomic by RT-qPCR. (A–N)** The expression of 14 key genes from each signaling pathway identified were tested by RT-qPCR from independent experiments performed in duplicate: uninjected, Control MO (ctrl MO) and *glra4a* MO. One-way ANOVA statistical analysis was performed (^∗^*p*-value < 0.05, ^∗∗^*p*-value < 0.01, ^∗∗∗^*p*-value < 0.0001).

In order to validate these transcriptomic results, we repeated the FACS purification on new independent clutches of embryos that we injected with control or *glra4a* morpholino, and tested the expression of 14 key genes (highlighted in yellow in **Figure 4**) involved in each of these five pathways by RTqPCR (**Figures 5A–N**). For all of these genes we found a positive correlation between the RNAseq data and the trend of expression observed by RTqPCR upon GlyR knockdown in NSCs. More importantly, for all these genes, we observed no difference in the expression between uninjected and control knockdown NSCs, thus confirming the specificity of our transcriptomic observations.

## Discussion

In this work, we aimed at identifying the molecular mechanisms involved in glycine-dependent neurogenesis during embryonic development, precisely in NSCs. By specifically looking into these cells after GlyR gene knockdown, we identified five signaling pathways that are very likely to play a role downstream to glycine. One of the most interesting pathways that we found is calcium signaling. Its involvement is consistent with our previous work in which we found that glycine-induced calcium transients in neuronal progenitors are required for neurogenesis (Brustein et al., 2013). Our present work suggests that calcium homeostasis is affected in NSCs upon GlyR knockdown and it identified specific members of this pathway as key molecular factors involved. Interestingly, although many proteins involved in intracellular calcium release (membrane calcium channels, ITPR1) as well as downstream effectors (CAMK4) were upregulated, the final targets of the pathway (ATF family transcription factors) were downregulated. This shows the complexity of the interpretation of such a large dataset. However, one could speculate that upon GlyR gene knockdown, calcium transients are disrupted in NSCs and that a compensatory mechanism is set up in order to rescue the entry of calcium into the cells. Moreover, it would be very interesting to identify among our dataset the secondary target genes that are likely to be misregulated because of the reduced expression of CREB complex members (AFT4B2, ATF7IP, ATF7A). These genes are normally regulated by these transcription factors and could be involved in NSC fate differentiation that would explain the specific loss of the interneuron population in the spinal cord.

Three other signaling pathways identified (TGF-beta, WNT, and SHH signaling) are also very good candidate since their implication in neurogenesis has been exhaustively reviewed (Aigner and Bogdahn, 2008; Mathieu et al., 2010; Faigle and Song, 2013; Varela-Nallar and Inestrosa, 2013; Urban and Guillemot, 2014). Indeed, the three of them are widely involved in embryonic and/or adult neurogenesis through modulation of cellular behaviors and their identification here helps confirm the usefulness of our progenitor FACS/RNAseq approach.

Bone Morphogenetic Proteins are the largest subgroup of the TGFs and they have been involved in numerous biological processes from cell survival and proliferation to differentiation in particular during the development of the central nervous system (Liu and Niswander, 2005). They have been characterized as inhibitors of neurogenesis during early development as they promote the development of ectoderm tissue at the expense of neuronal fate (Wilson and Hemmati-Brivanlou, 1995). Furthermore, both noggin-mediated BMP inhibition and chemical BMP inhibition have been shown to promote proliferation of NSCs and therefore neurogenesis (Guo et al., 2011; Neely et al., 2012). However, the role played by BMPs is more complex in the adult brain where their blockade leads to the depletion of the stem cell niches, suggesting a role of BMPs in the maintenance of stemness and therefore of long-term adult neurogenesis (Mira et al., 2010). Our analysis indicates that BMP2B, BMP4 and BMP6 are all three upregulated in NSCs upon GlyR knockdown (**Figures 5G,H**). According to the literature, this finding would be consistent with an inhibition of neurogenesis. Moreover, SMAD3 transcription factor, a target of TGFs that is also a crucial player promoting neurogenesis in adult mice (Wang and Symes, 2010), is downregulated in our dataset (**Figure 5I**). Altogether, these results suggest that the abnormal neurogenesis induced by GlyR knockdown could be, at least in part, due to misregulation of TGF-related proteins.

Sonic HedgeHog signaling is important for the patterning of the dorso-ventral neuron types within the developing neural tube where it promotes ventral identities (Rowitch and Kriegstein, 2010). Therefore, its modulation could more likely be involved in a shift in cell fate. Although we did not previously observe that the lack of interneuron population upon GlyR blockade was made at the expense of another neuronal cell population (Cote and Drapeau, 2012), we can speculate that SHH signaling could be involved in the differentiation process of this specific interneuron population. Indeed, SHH signaling has been reported as modulating NSC proliferation and therefore the generation of newborn olfactory interneurons from the adult sub ventricular zone (SVZ) in mammals (Palma et al., 2005).

WNT signaling was shown as positively regulating neurogenesis in mammalian embryos (Kalani et al., 2008). Not only is it required for the generation of different types of neurons, but it also plays a role in the self-renewal properties of NSCs. Two inhibitors of the WNT pathway (WIF1 and DKK1B) were upregulated upon GlyR knockdown (**Figures 5K,L**), thus allowing us to assume that Wnt-dependent neurogenic processes could be silenced in NSCs when glycine signaling is impaired. Consistently, it has been reported that WNT inhibition through DKK1 overexpression prevents neuronal production during mouse embryonic neurogenesis (Munji et al., 2011). Moreover, the downstream target FOSL1A (upregulated in our dataset, **Figure 5M**) is a transcription factor regulating the expression of a broad panel of genes, some of them being necessary for cell proliferation although in endothelial cells (Evellin et al., 2013).

Even more interestingly, interactions between these signaling pathways have been recently shown to be requisite for stem cell differentiation. In fact, WNT and SHH pathways act downstream of TGF signaling to promote dopaminergic neuronal differentiation *in vitro* (Cai et al., 2013). Altogether, these data indicate WNT, SHH, and TGF-beta signaling as top candidate pathways involved downstream of glycine to promote neurogenesis, though the molecular mechanisms involved could be more complex through the integration of multiple canonical pathways.

Lastly, our gene expression analysis identified P53 as being upregulated specifically upon GlyR knockdown (**Figures 4** and **5N**). Interestingly, P53 activation has been recently shown as regulating neurogenesis by increasing proliferation and differentiation of hippocampal NSCs *in vitro* (Grigor’eva and Glazova, 2015). However, P53 is better known as a pro-apoptotic factor, suggesting that NSCs might die in the absence of glycine signaling. Our previous work did not detect an increase in cell death in the developing spinal cord of morphants at 48hpf (McDearmid et al., 2006), although it could occur at the earlier stage examined here (20 hpf). P53 is often upregulated after injection of morpholinos, but our 5 base-mismatched control morpholino injection did not show any upregulation of p53 (**Figure 5N**). As a result, more discrete cell death could arise in a subpopulation of neural progenitors in the developing spinal cord.

The next step in deciphering the molecular aspects of glycine-dependent neurogenesis will be to perform functional genomics on the candidate genes we identified. Such a characterisation is beyond the scope of the present work as a large effort needs now to be deployed to understand the function of these molecular actors. Zebrafish would be a suitable model for such an analysis since the embryo is easily genetically accessible. Lastly, our present work widens the use of zebrafish as a model of developmental biology. Indeed, we took advantage of advanced cell purification techniques to precisely look within our cells of interest but without dismantling the *in vivo* context in which cells develop. As a result, zebrafish embryos can be considered as *in vivo* test tubes in which it becomes more and more easy to dig for molecular characterization of phenotypes.

## Author Contributions

AB validated the *in vivo* morpholino knockdown. AB and ES designed FACS experiments. ES performed and interpreted the transcriptomic and pathway analyses and did RTqPCR validations. ES and PD wrote the manuscript.

## Conflict of Interest Statement

The authors declare that the research was conducted in the absence of any commercial or financial relationships that could be construed as a potential conflict of interest.

## Supplementary material

TABLE S1 **Differentially expressed genes upon GlyR knockdown in zebrafish embryo NSCs.** By filtering with a |log2FC| > 1 and a *p*-value < 0.05, we found 1,162 genes differentially expressed upon GlyR knockdown specifically in NSCs. 896 are upregulated (green up arrow) and 266 downregulated (red down arrow). For each gene, the log2FC, the p value and the base mean values from the DESeq2 analysis are provided. Genes highlighted in blue are those that have been taken back from the overlapping genes also found in control knockdown (see test#3, **Figure 3A**).
